# Automated Parameter Extraction Of ScAlN MEMS Devices Using An Extended Euler–Bernoulli Beam Theory

**DOI:** 10.3390/s20041001

**Published:** 2020-02-13

**Authors:** Maximilian Krey, Bernd Hähnlein, Katja Tonisch, Stefan Krischok, Hannes Töpfer

**Affiliations:** 1 Advanced Electromagnetics Group, Department of Electrical Engineering and Information Technology, Technische Universität Ilmenau, Postfach 100565, 98684 Ilmenau, Germany; hannes.toepfer@tu-ilmenau.de; 2 Technical Physics 1 Group, Institute of Micro- and Nanotechnologies (IMN MacroNano^®^), Technische Universität Ilmenau, Postfach 100565, 98684 Ilmenau, Germany; bernd.haehnlein@tu-ilmenau.de (B.H.); katja.tonisch@tu-ilmenau.de (K.T.); stefan.krischok@tu-ilmenau.de (S.K.)

**Keywords:** MEMS, scandium aluminium nitride, magnetoelectric sensor, Young’s modulus, automation, algorithm

## Abstract

Magnetoelectric sensors provide the ability to measure magnetic fields down to the pico tesla range and are currently the subject of intense research. Such sensors usually combine a piezoelectric and a magnetostrictive material, so that magnetically induced stresses can be measured electrically. Scandium aluminium nitride gained a lot of attraction in the last few years due to its enhanced piezoelectric properties. Its usage as resonantly driven microelectromechanical system (MEMS) in such sensors is accompanied by a manifold of influences from crystal growth leading to impacts on the electrical and mechanical parameters. Usual investigations via nanoindentation allow a fast determination of mechanical properties with the disadvantage of lacking the access to the anisotropy of specific properties. Such anisotropy effects are investigated in this work in terms of the Young’s modulus and the strain on basis of a MEMS structures through a newly developed fully automated procedure of eigenfrequency fitting based on a new non-Lorentzian fit function and subsequent analysis using an extended Euler–Bernoulli theory. The introduced procedure is able to increase the resolution of the derived parameters compared to the common nanoindentation technique and hence allows detailed investigations of the behavior of magnetoelectric sensors, especially of the magnetic field dependent Young‘s modulus of the magnetostrictive layer.

## 1. Introduction

For many applications of miniaturized MEMS, aluminum nitride (AlN) has become a standard material [1,2]. Only recently, scandium aluminum nitride has been proposed as an efficient expansion of the nitride system providing an enormous increase in the piezoelectric constants [3,4]. Typical microelectromechanical systems (MEMS) application can be found in the field of radio frequency (RF) filters [5], micro actuators [6], and energy harvesting devices [7].

Especially in the field of MEMS-based magnetoelectric sensors, major advances have been made recently, since, for the detection of magnetic fields in the pico tesla range at room temperature, magnetoelectric sensors (MES) are a promising technology [8,9,10] with possible applications in bio-medicine [11,12] and non-destructive testing [13]. Such a sensor correlates a magnetic input to an electric output signal, and the quotient of in- and output is the magnetoelectric coefficient. A high magnetoelectric coefficient is achieved by designing a composite of magnetostrictive and piezoelectric layer in one sensor [14] and driving the sensor at its mechanical resonance [15]. Thus, we will use a typical multilayered beam structure as used in magnetoelectric MEMS-based sensor devices as sample structure.

MES sensors are usually designed as a singly or doubly clamped beam in a wide range of sizes between 100 μm [16] and several centimeters [17], while this work is focusing on smaller (integratable) structures. On the one hand, in the presence of a magnetic field, the magnetostrictive layer either expands or shrinks, depending on the specific magnetoelastic properties of the material. As a consequence, the applied tension leads to a change of the eigenfrequency of the beam due to bending or induced strains in singly and doubly clamped beams, respectively. On the other hand, the magnetostrictive material decreases also its Young’s modulus by up to 30%, depending on the intensity of the magnetic field [18]. This behavior is called ΔE effect. As the induced stresses and the ΔE effect act on the device performance at the same time, both influences have to be distinguished correctly from each other. Hence, weak fields are challenging to detect. The eigenfrequency shift can be detected via the voltage generated by the piezoelectric layer. Sensors based on a eigenfrequency-shift-principle are known to have high sensitivity and are robust to intensity fluctuations [19,20]. Due to size and a principle based on vibrations, such a sensor is categorized as a MEMS.

Since small eigenfrequency shifts are expected in the presence of weak magnetic fields, well suited models to detect the shifts as well as for parameter extraction are required. We propose a model that accommodates not only for eigenfrequencies but the vibrational behavior in a broader sense: a complex fit to the FFT (fast Fourier transform) of the beam vibration. This way, all measured harmonics are included as well as the shape of their peaks. The latter is interesting for future investigations regarding damping. An adequate fit function is derived in Section 3.

The performance of a sensor is affected by many influences, such as: material constants, ambient conditions, geometry of the beam and its layers and the magnetic flux density. Simulations have to include the different fields of physics such as the mechanic, electric and magnetic domain as well as the multiferroic character of their interactions [21,22]. On top of that, a multi parameter problem arises due to many degrees of freedom in materials and geometric design. This creates potential for optimization where two targets are important: high sensitivity to the magnetic field and big amplitude of the output signal.

To optimize these targets, the choice of appropriate materials is important. Hence, for the piezoelectric layer, materials with a high piezoelectric constant are of special interest, like scandium aluminium nitride (ScAlN) [8,23]. It combines several advantages like easy deposition and CMOS integration [24], high temperature stability [25] and is in contrast to PZT, lead free. Analogous, for the magnetostrictive layer, materials with a high magnetostrictive constant are needed. Cobalt iron (Co/Fe) multilayers or Co-Fe alloy films satisfy this condition [26]. To combine materials with these characteristics is suggested by [10]. Since its material properties change with size and crystal orientation, they have to be investigated for desired scale and orientation. This work deals with the implementation of an automated process of the extraction of material properties from measurement data to feed simulations and models in general. From the vibration behavior of cantilevers (clamped on one side) and bridges (clamped on both sides), thus structures of the same material the Young’s modulus can be determined [27] as shown in Section 6. The Euler–Bernoulli beam theory also known as classical beam theory is used to design this process. The beam theory is modified for modal analysis of slender beams exhibiting inner strain [28] as well as curvature [29] of the beam and is thus denoted as extended Euler–Bernoulli Theory (EEBT). The EEBT is utilized to investigate the desired parameters, namely the Young’s modulus and the strain dependent on the orientation. Using this analytical method, we present a fast and precise algorithm with explicit equations and without the need of time-consuming finite element calculations.

## 2. Measuring Samples and Experimental Setup

**Figure 1:**
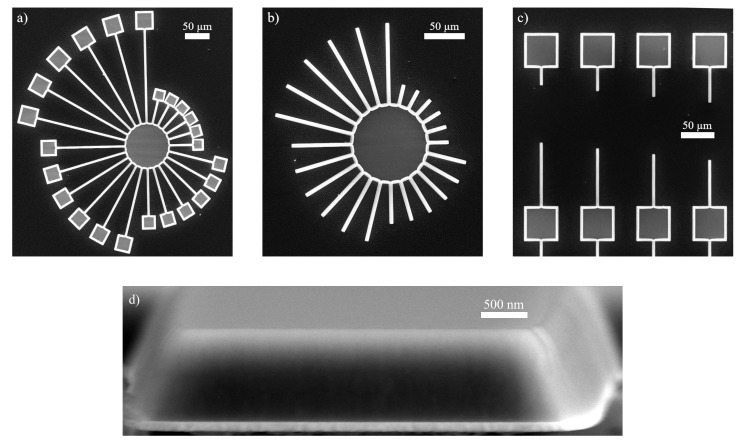
SEM images of ScAlN MEMS structures directly on silicon with well distinguishable undercut regions. (**a**) doubly clamped bridges (50 … 200μm) and (**b**) singly clamped cantilevers (25 … 100μm) in star configuration with an angle delta of 15${}^{{}^{\circ}}$; (**c**) cantilevers in harp configuration of different lengths and constant orientation; (**d**) cross section of a cantilever consisting of Pt (80 nm)/ScAlN (1μm) with trapezoidal shape after etching.

The piezoelectric scandium aluminium nitride layer with a scandium amount of 8 at.-% was grown using a co-sputter process at 300 ${}^{{}^{\circ}}C$ on a Ti(111)/Pt(111)/Si(111) substrate. The thickness of the titanium, platinum and the ScAlN is 20 nm, 80 nm and 1 μm, respectively. ScAlN exhibits a strict c-axis orientation [30] with an in-plane rotation of the individual pillars according to X-Ray diffraction measurements. The structuring of the sample was carried out using e-beam lithography and different (an-)isotropic dry etching techniques, namely chlorine plasma for the ScAlN, argon plasma for Pt and finally fluorine plasma for suspending. The structure design consists of differently oriented MEMS in a star and harp layout (see Figure 1). In the following, harps and stars are referred to as h- and s-structures. The structures shown in the figures a to c are without Ti/Pt between ScAlN and silicon to be able to demonstrate the undercut regions that are needed for the latter analysis. All structures have the same width (5.3 μm) and are varied in length. The s-bridges exhibit lengths between 50 and 200 μm and the s-cantilevers between 25 and 100 μm. For a more accurate investigation of the length, the h-layout consists of eleven beams with lengths between 20 and 220 μm for the h-bridges and between 20 and 120 μm for h-cantilevers, respectively. After the etching, different cross sections of the MEMS appear. ScAlN shows a trapezoidal shape, whereas the ones made of Pt have a rectangular shape. This is shown in Figure 1d. Titanium is etched in fluorine plasma and hence not considered in the latter analysis.

A Polytec UHF-120 Laser Doppler Vibrometer (LDV) (Polytec GmbH, D-76337 Waldbronn, Germany) is used to measure the mechanical vibration of the microbeams vs. time and conversion to frequency domain (FFT spectrum). The FFT spectrum consists of 12,800 lines between 50 kHz and 4 MHz. The used laser power is 5 mW at 532 nm. To avoid thermal shifts due to the laser energy during measurement, only single-point spectra are measured. A single-point spectrum observed at a vibration node lacks the peak of the corresponding eigenfrequency, which is why the data are recorded close to a clamping, where only nodes of high order eigenfrequencies are expected. Shortest bridges and longest cantilevers could not be measured as their eigenfrequencies were outside the measurement limits. Every beam length and orientation is represented multiple times, for measurement certainty.

**Figure 2:**
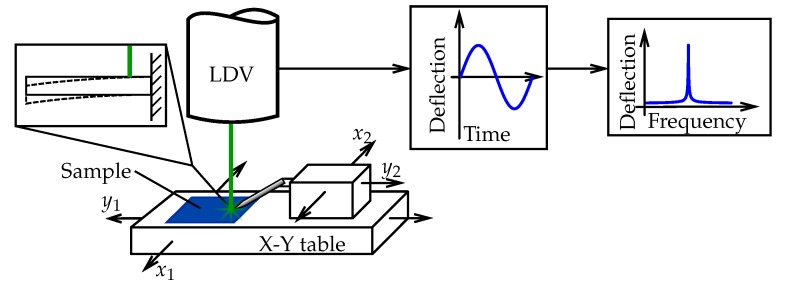
Measuring setup. The *x*–*y* table moves the blue sample relative ($x_{1},y_{1}$) to the stationary Laser Doppler Vibrometer (LDV). The tungsten tip for excitation moves with the sample but can actuate independently ($x_{2},y_{2}$) to a desired structure.

**Figure 3:**
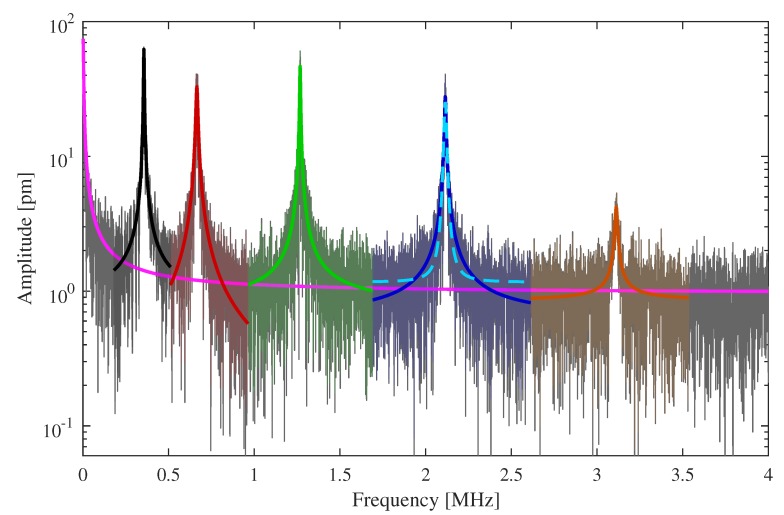
Frequency spectrum Λ obtained from a 75μm long cantilever of a s-layout by the LDV (gray line). The curve fit of the asymptotic part is colored magenta. The dashed cyan colored line represents an exemplary fit to the data with the widely used Lorentzian function. The other colored lines show the individual fits with the function derived in Section 3 of the *n* peaks and their fit range.

A sketch of the measurement setup is shown in Figure 2. The sample is placed on a cross table under the LDV. Aided by the LDV’s microscope, the desired beam is positioned to the laser dot. The beams’ vibrations are induced via electrostatic excitation with a tungsten tip and a multi carrier continuous wave signal. The signal is produced by a Rohde & Schwarz SMBV100A signal generator (Rohde & Schwarz GmbH & Co. KG, D-81671 München, Germany) with a voltage of 1 V and is amplified with a Ciprian US-TXP-3-C amplifier (CIPRIAN Sarl, FR-38330 Saint Ismier, France) by a gain of 200. The tungsten tip has a diameter of about 10 μm and is placed about 100 μm above the sample. Because of the tip, a certain error arises from the experimental setup, leading to an inhomogeneously distributed electric field across the MEMS and thus to an unsteady load. As the total deflection of the structures is always below 1 nm, we assume the influence and any other nonlinear effects on the vibrational behavior to be negligible. In the following, the FFT data Λ obtained from the LDV is used for analyzing the vibration behavior. Figure 3 shows an example of a raw spectrum from the LDV with the gray line. This and further raw spectra are available as Supplementary Materials for this paper.

Overall, 322 beams on the sample were measured. On average, a beam spectrum shows about five eigenfrequencies. Manual parameter extraction takes for each sample approximately ten days. To accelerate this process, an automation is reasonable. Core of the automation process is a curve fit on the FFT spectrum.

## 3. Modeling the Spectrum

First, a model for the complex vibrational behavior is needed. Lorentzian functions derived from the vibrational behavior of charged particles are often used to model a peak of the frequency response [31,32]. However, this function is not able to represent the $\frac{1}{x}$-like slope on both sides of the maximum adequately, as shown in Figure 3 with the cyan colored dashed line. Using the Lorentzian function instead of the subsequently proposed fit function leads to errors in the determined parameters. According to the fit in Figure 3, the error of the eigenfrequency is negligible, but for amplitude and full width half maximum respective errors of 13% and 26% can be calculated for this example. Even higher deviations are observable for other structures. This is directly affecting derived values, like the quality factor as a main characteristic for the description of MEMS. Hence, the model we introduce is based on mechanical vibrations. The time dependent vibration g(t) of a damped mass point can be represented by the following equation:

(1) $$g\left(t\right)=a_{n}e^{j\;2\pi f_{n}t}\;\cdot \;e^{-p_{n}t},$$

where $a_{n}$ is the amplitude parameter, *t* the time, $f_{n}$ the eigenfrequency, $p_{n}$ the damping coefficient, and *j* the imaginary unit.

A frequency peak is modeled by performing a Fourier transform of Equation (1)

(2) $$\tilde{G}\left(f\right)=\frac{1}{\sqrt{2\pi }}\int_{-\infty }^{\infty }g\left(t\right)e^{-j\;2\pi ft}dt,$$

where *f* denotes the frequency. By calculating the absolute value $\Gamma \left(f\right)=|\tilde{G}\left(f\right)|$, every peak can be represented by the spectral function

(3) $$\Gamma \left(f\right)=\frac{a_{n}}{\sqrt{p_{n}^{2}+{\left(f-f_{n}\right)}^{2}}}+\theta_{n}.$$

The parameter $\theta_{n}$ is added to include offsets of the fitted spectra. Thus, four parameters ($a_{n}$,$p_{n}$,$f_{n}$ and $\theta_{n}$) have to be fitted for every peak *n*. In Equation (3), $f_{n}$ marks the middle of the peak with the amplitude given by

(4) $$Z_{n}=\frac{a_{n}}{p_{n}}+\theta_{n}.$$

The full width half maximum (FWHM) $\Delta f_{n}$ can be found using Equation (4) by solving the following equation

(5) $$\Gamma \left(\Delta f_{n}\right)=\frac{Z_{n}}{2},$$

which leads to

(6) $$\Delta f_{n}=2\sqrt{3}\;p_{n}.$$

Even though the vibration is forced, no factor that considers the time dependent force is implemented in Equation (1). Assuming the force would be sinusoidal, a phase shift as well as a change in amplitude arises. Both effects can be omitted in our model. The amplitude is a parameter for the fit, changing it has no effect on the desired target of the minimization procedure in Equation (7). A phase shift could affect the relation between driving force and the vibration, while having no influence on the shape of the peak.

In total, this modelling function derived from the time dependent vibration of a mass point allows to fit eigenfrequency peaks in all of their properties, namely peak position, amplitude, and FWHM. Thus, this function is used throughout this paper.

## 4. Curve Fitting Algorithm

**Figure 4:**
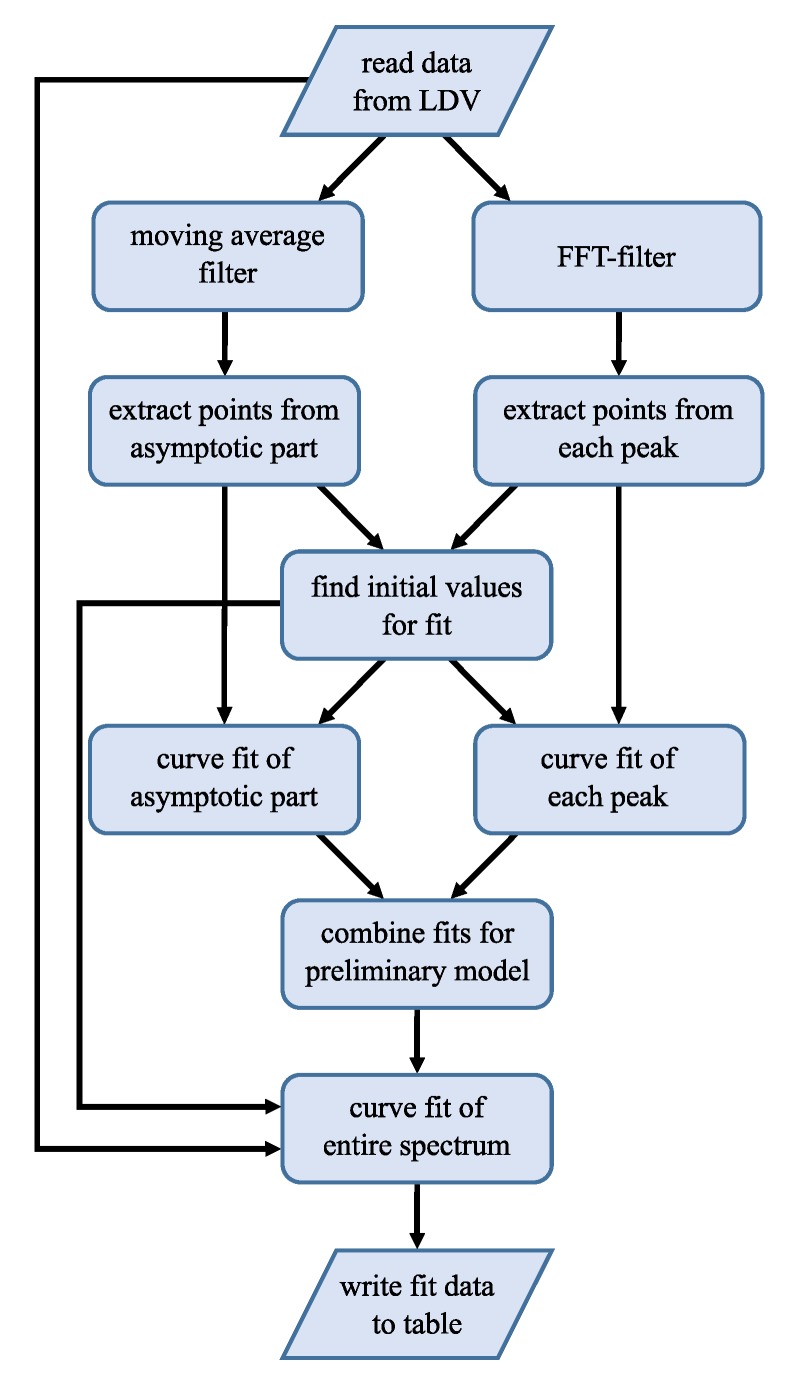
Flowchart for the fitting algorithm for one spectrum from the LDV raw data to the fitted spectrum. The generated data is used for further processing (see Figure 6).

The applied algorithm is outlined in Figure 4 and realized with MATLAB [33]. In the first step, the data is read from the LDV. Then a fit of the asymptotic part of the FFT close to the *x*-axis as shown in Figure 3 is needed. For this fit, only the data close to the *x*-axis as well as the minima between the peaks are used. A moving average filter is used to smooth the data. A deviation is calculated with the squared distance of the spectral function (3) to the data points $\Lambda_{asym}$. Using MATLAB’s multi-variable optimization function fminsearch, a curve fit is performed to minimize the deviation defined as

(7) $$\sum_{i=1}^{M}{\left(\Lambda_{asym}^{i}-\Gamma_{asym}^{i}\right)}^{2}\overset{}{\to }\min,$$

with *M* being the number of data points. Both $\Lambda_{asym}$ and $\Gamma_{asym}$ are obtained at the same frequencies $f_{asym}$. Appropriate initial values for the optimization, in the following marked with the index iv, were derived from the data. The initial value of the offset $\theta_{n,iv}$ is chosen to be 0. By assuming that the peak of $\Gamma_{asym}$ is close to 0, $f_{n,iv}=0$ is set. Since Equation (6) connects the damping coefficient $p_{n}$ with the FWHM $\Delta f_{n}$, it can be calculated from the data. An interpolation to find the value of $f_{h,n}$ where $\Lambda_{asym}\left(f_{h,n}\right)=\frac{Z_{n}}{2}$ is done, assuming the maximum of $\Lambda_{asym}$ being equivalent to amplitude $Z_{n}$ in Equation (4). Doubling $f_{h,n}$ is set to be the initial value for the FWHM and, with (6), $p_{n,iv}$ is calculated. Parameter $a_{n,iv}$ is then calculated via Equation (4) with $p_{n,iv}$ and the $Z_{n}$ from $\Lambda_{asym}\left(f\right)$. Then, the optimization process is executed and as a result we receive $\Gamma_{asym,opt}$.

**Figure 5:**
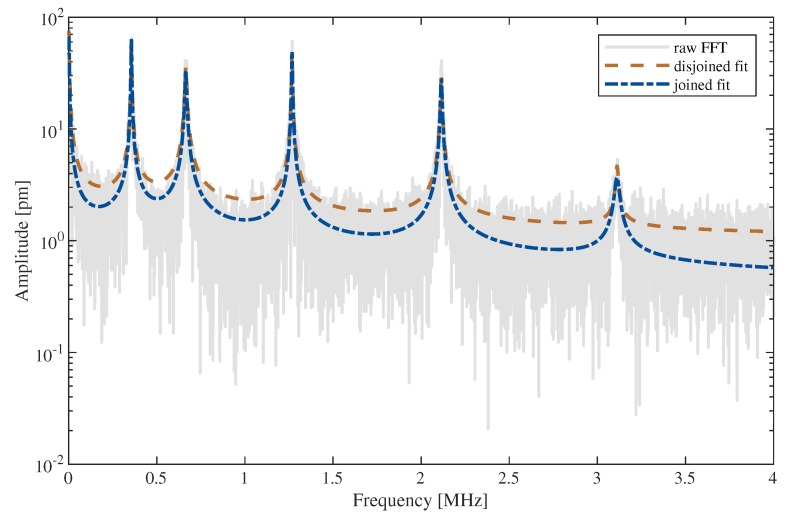
Comparison between the joined and disjoined fit. The joined fit is more accurate with respect to the raw FFT obtained from the LDV.

In the next step, each peak will be fitted separately with Equation (3). To reduce noise, a FFT is applied to an over-sampled version of the raw FFT data and the high frequency parts are subtracted, which effectively implements a low pass filter. In Figure 4, this is called ‘FFT-filter’. First, the peaks height $Z_{n}$, frequency $f_{n}$ and $\Delta f_{n}$ are extracted from the data using the MATLAB findpeaks function, which also delivers the peak FWHM. Then, the range $\Lambda_{p,n}$ around each peak *n* is defined. The ranges do not overlap and the fit procedure is performed merely on $\Lambda_{p,n}$ and its corresponding frequencies $f_{p,n}$ as shown in Figure 3 by the colored lines. Initial values are again obtained from the data. The offset $\theta_{n,iv}$ is the mean of the first and last point of $\Lambda_{p,n}$. The peak frequency $f_{n,iv}$ is directly passed from the findpeaks function, $p_{n,iv}$ is calculated via Equation (6) by using the FWHM $\Delta f_{n}$ from the findpeaks function. The initial value of $a_{n,iv}$ depends on the amplitude from findpeaks as well as $p_{n,iv}$ and $\theta_{n,iv}$; see Equation (4). The optimization is then done for each peak with individual initial values. We obtain $\Gamma_{p,n}$ as shown in Figure 3 by the colored curves. A preliminary model for the spectrum is given by

(8) $$\Gamma_{pre}=\Gamma_{asym,opt}+\sum_{n=1}^{N}\Gamma_{p,n},$$

where *N* is the total number of peaks. It is shown in Figure 5 as disjoined fit. The goodness of fit (GoF) is calculated for every peak using the normalized root mean square error (NRMSE). Since errors occur, for example due to too noisy data or undetected small peaks, the GoF can be used as a simple indicator for the usability of the generated model.

The last step of the curve fitting algorithm is to fit the full spectrum. This is done on raw FFT data as obtained from the LDV. In (8), the separated fits with the offsets of every peak and the asymptote are summed up. This leads to too high valleys between the peaks. In Figure 5, the red line should be roughly in the middle of the noisy gray line. By performing a fit of the preliminary model (8) to the raw data, with the already optimized parameters as initial values for this final fit, the blue curve in Figure 5 is obtained. Now, not only four parameters are fitted but 4+4N. However, with good initial values, convergence is reached fast.

Initial values, optimized parameters, GoF as well as raw data and fitted model data are saved for every spectrum in one line of a table within a MATLAB structure for further processing.

## 5. Fit Result Discussion

On average, a single spectrum contains five to six peaks, and it takes about 23 s to perform the fit outlined in Section 3, including an export of a figure similar to Figure 3 for debugging and observation purposes. Fitting all 322 spectra of one sample takes about 1.2 h computing time without human intervention.

The fit is of lesser accuracy, when the peaks are close to each other; this can be seen in Figure 5. The blue line between the first and second peak is on the upper end of the raw FFT. Overlapping of the fit functions of each peak causes this error. However, as shown in [31,34] and [35], measurements under low air pressure lead to significantly better quality factors and smaller FWHMs. Because of the high sensitivity to pressure, the authors of [36] suggest using such beam resonators for pressure measurement. The peak overlapping would be reduced with higher quality factors.

The initial as well as the optimized values of the two-stage curve fit are shown in Table 1; they belong to the fit of the first eigenfrequency of the spectrum shown in Figure 3 and Figure 5. The parameters $a_{n}$ and $p_{n}$ get smaller in both steps; they seem to converge to a minimum. The eigenfrequency of the peak $f_{n}$ stays almost constant over both optimizations; this is expected since a shift in frequency will definitively increase the deviation value in Equation (7). The offset $\theta_{n}$ decreases in the first step, but is increased in the final step. This behavior is probably due to the closeness of the first and second peak as mentioned above. The disjoined fit uses merely the data close to the peak. The joined fit considers the whole spectrum, thus close peaks affect the parameters of each other. In particular, their offset is influenced because of the overlapping as previously remarked.

To check the obtained parameters and the algorithm in general, the optimized $p_{n}$ value of Table 1 can be compared to the FWHM obtained from the joined fit. From Figure 5, we determine a FWHM of 6.0388 kHz for the first peak. Using Equation (6), the corresponding damping coefficient is calculated to $p_{1,join}=1.7433\;k\mathrm{Hz}$, which differs by 0.67% from the value in Table 1. A small deviation is expected because of the influence of the peaks’ parameters to each other in the joined fit. In view of the detection of small eigenfrequency shifts due to the magnetostrictive sensor principle, a high accuracy of the determined FWHMs and quality factors is essential.

With a GoF under 40%, the fit is considered failed, which usually happens on very noisy data. This occurs on about 7.2% of the measured spectra. The GoF of the remaining data is on average 71.8± 11.5%. Please note that, due to the noise, a GoF of 100% is neither possible nor desired.

## 6. Modal Analysis

The EEBT is a special case of the Timoshenko beam theory [37] that allows an analytic approach for the determination of the mechanical parameters. The preconditions for its application are small deflections during excitation and string-like structure aspect ratios, which means width and height of the beam are much smaller than its length [38]. Otherwise, the Kirchhoff–Love plate theory [39] should be used to consider longitudinal and transversal bending-mode splitting. As stated in the Introduction, the EEBT describes the vibrational behavior of curved cantilevers and strained bridges. For each structure, the eigenfrequencies $f_{n}$ of mode number *n* can be determined using [40]

(9) $$f_{n,c}=\frac{\kappa_{n,c}^{2}-\alpha^{2}}{2\pi l_{c}^{2}}\sqrt{\frac{{\left(EI\right)}_{tot}}{{\left(\rho A\right)}_{tot}}}$$

and

(10) $$f_{n,b}=\frac{\kappa_{n,b}^{2}}{2\pi l_{b}^{2}}\sqrt{\frac{{\left(EI\right)}_{tot}}{{\left(\rho A\right)}_{tot}}}\sqrt{1+\gamma_{n}\frac{l_{b}^{2}}{h^{2}}\epsilon_{b}},$$

where indices *c* and *b* denote cantilevers and bridges, respectively. The eigenvalue $\kappa_{n}$ is depending on the mode and the boundary condition [28,41]. The bending stiffness ${\left(EI\right)}_{tot}$ is given by the Young’s modulus *E* and the moment of inertia *I*. The reduced mass ${\left(\rho A\right)}_{tot}$ includes the density ρ and the cross section *A*. Bridges loaded with the strain $\epsilon_{b}$ are described by the second mode dependent eigenvalue $\gamma_{n}$. This eigenvalue is strain-dependent according to the numerical calculations presented in [42]. However, only the first eigenvalue is derived in this work and thus cannot be used solely on the analyzed data set with included higher eigenmodes. As a consequence, $\gamma_{n}$ is assumed to be constant for the present analysis. Recent investigations of 3C-SiC (111) bridges [43] showed that $\gamma_{n}$ tends to be constant even for higher strains. As this finding is not directly applicable to ScAlN bridges, separate studies for the present material system are needed. According to [42], the arising error is approximately 1% for the highest determined strains and decreasing for decreasing strains. The parameter *h* stands for the total thickness of the MEMS (Pt and ScAlN). The cantilever curvature α is neglected in our structures as the out-of-plane stress gradient of the hetero-layer Pt/ScAlN is small compared to the in-plane stresses. Since each layer of the hetero-structure has its own unique properties, Equations (9) and (10) are extended by:

(11) $${\left(EI\right)}_{tot}=\sum_{i}E_{i}\left(I_{i}+a_{i}A_{i}\right),\;a_{i}=y_{s,i}-y_{s},$$

(12) $$y_{s}=\frac{\sum_{i}\left(h_{i-1}+y_{s,i}\right)A_{i}E_{i}}{\sum_{i}A_{i}E_{i}},$$

(13) $${\left(\rho A\right)}_{tot}=\sum_{i}\rho_{i}A_{i}.$$

It should be noted that, due to the change in the neutral axis $y_{s}$, Equations (9) and (10) become quite complex when the layer count increases.

As MEMS with two different boundary conditions are used (singly or doubly clamped), Equations (9) and (10) allow to uniquely determine two parameters. In the present case, these are the Young’s modulus of the ScAlN layer and the strain of the bridges. Hence, the other properties have to be measured beforehand. The Young’s moduli of the surrounding metallic layers have to be measured for each layer separately.

**Figure 6:**
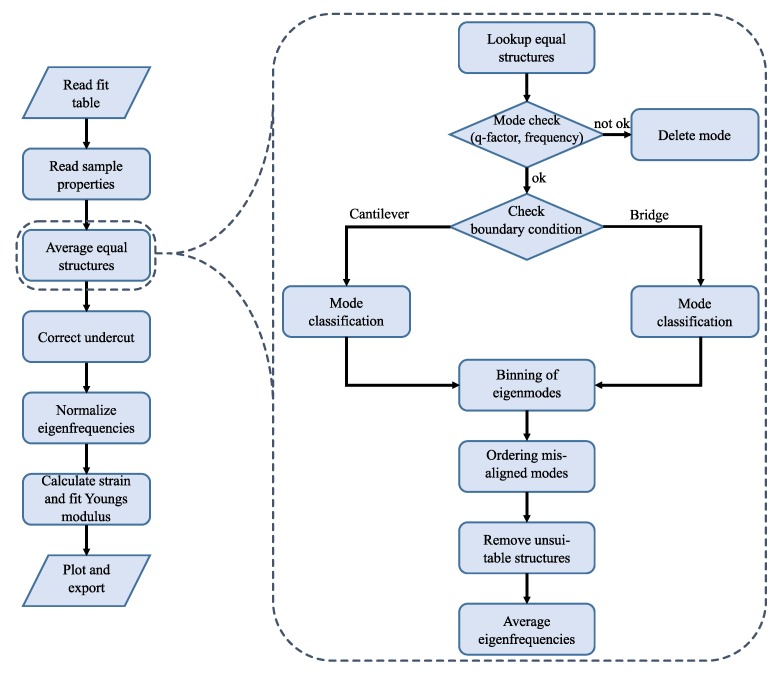
Subsequent flowchart for the analysis and extraction of the mechanical parameters after fitting the frequency spectrum. The critical step for averaging equal structures is shown in a more detailed view.

The necessary steps for the analysis of the fitted eigenfrequencies using the EEBT are shown in the flowchart in Figure 6. First, the table with the fit parameters for the whole sample discussed in the previous section is imported. Afterwards, the sample information is retrieved that is stored in a separate function file. The information consists of:

- Young’s moduli of the surrounding layers (e.g., Ni, Pt, none),
- densities of the used materials,
- structure count and respective boundary condition (c, b),
- geometrical aspects and orientation.

In the following step of “averaging equal structures”, the table is scanned for MEMS of the same geometrical aspects and boundary conditions (up to four s- and six h-structures by design). The found eigenfrequency vectors are then checked for consistency. Erroneous fitting results like diverged peaks with unexpected FWHM (or quality factor), GoF or eigenfrequency are deleted, accordingly. Depending on the boundary condition, the different eigenfrequencies have to be assigned to the respective eigenmode. This is a critical task as it is unknown whether the first eigenfrequency is also the first eigenmode, for example in the case when the natural mode was below the measurement limit or undetected during the spectrum fit. Furthermore, it is possible that some higher harmonics are missing in the frequency vector due to measuring only the single point spectrum at a node. Additionally, some structures could exhibit torsional modes in their spectrum that also have to be considered. Therefore, a robust algorithm is needed for a proper mode classification. Otherwise, a correct averaging is not possible.

Within a single structure, most of the parameters in Equations (9) and (10) are constant, except $f_{n}$ and $\kappa_{n}$. Thus, a determination method that is only relying on the eigenfrequencies of the same structure is a convenient way to exclude uncertainties (for example the undercut that is discussed later). In consequence, the ratio of eigenfrequencies is used to determine the correlation between the ratio of eigenvalues and finally to assign the eigenmode. In the case of unbent cantilevers, the difference between both ratios has to be minimized:

(14) $$\left|\frac{\kappa_{n,c}^{4}}{\kappa_{i,c}^{4}}-\frac{f_{n,c}^{2}}{f_{i,c}^{2}}\right|\overset{}{\to }\min,$$

where i={1,…,n−1},i<n. The minimization problem leads to n−1 matrices from whom the positions of the minima are directly indicating the respective eigenmodes. For strained bridges, it was found that an adjustment to the theoretical eigenvalue ratio has to be done for better mode classification, namely

(15) $$\left|\frac{{\left(\kappa_{n,b}-k_{b}\right)}^{4}}{{\left(\kappa_{i,b}-k_{b}\right)}^{4}}-\frac{f_{n,b}^{2}}{f_{i,b}^{2}}\right|\overset{}{\to }\min,$$

where $k_{b}$ as an empiric constant presumably related to the deviations of real bridges from the EEBT (e.g., complex strain distribution). Best minimization results were achieved for $k_{b}\;\approx \;0.14$. However, after such mode assignment, the full consistency is usually not reached. The reason is the mentioned torsion modes and slight eigenfrequency shifts which can lead to a wrong classification. For example, considering four equal structures of an exemplary 75 μm long s-cantilever, the following eigenfrequency matrix was determined:

(16) $$\left[\begin{array}{ccc} 0.2202 & 1.3744 & 0\\ 0.2202 & 1.3703 & 0\\ 0.2195 & 1.3711 & 0\\ 0.2337 & 0 & 1.3702 \end{array}\right]\times 10^{6}\;\mathrm{Hz}.$$

It can be easily noted that the second eigenmode of the fourth structure is wrongly classified as the mode three. Solving this issue is possible by binning the eigenfrequencies based on the histogram. However, because the bin size is not constant, it has to be adjusted in accordance to Equations (9) and (10). For cantilevers, the eigenfrequencies scale with $\kappa_{n,c}^{2}$ and so the bin size must too. For strained bridges, the situation is a bit different. The eigenvalues $\kappa_{n}$ and $\gamma_{n}$ behave inversely proportional to each other, i.e., depending on $\epsilon_{b}$ the scaling of the eigenmodes changes. It was found that a bin size scaled by the power of 1.5 is able to cover the shifting eigenfrequencies very well. With the proposed correction, the eigenfrequencies are finally well averaged over the redundant structures.

The correct mode classification allows in the next step the investigation of the undercut influence. It is a consequence of the isotropic etching and not covered by the EEBT. The existence of the undercut results in a red shift of the eigenfrequencies due to an effective increase of the cantilever length. This increase is mainly driven by the tips that are formed under the beam support as can be seen in the scanning electron microscopy (SEM) images in Figure 1b,c. These tips are usually smaller than the absolute undercut length. This deviation from a string like cantilever leads to an error in the determination of the true cantilever eigenfrequencies.

**Figure 7:**
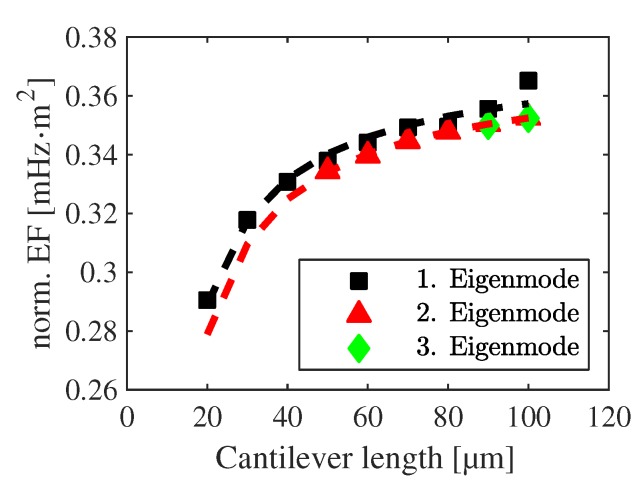
Experimentally observed red shift of the cantilever eigenfrequencies after normalization by length and eigenvalue. The fit function is used to extract the influencing undercut for $l\;\overset{}{\to }\;\infty$ during resonant excitation.

**Figure 8:**
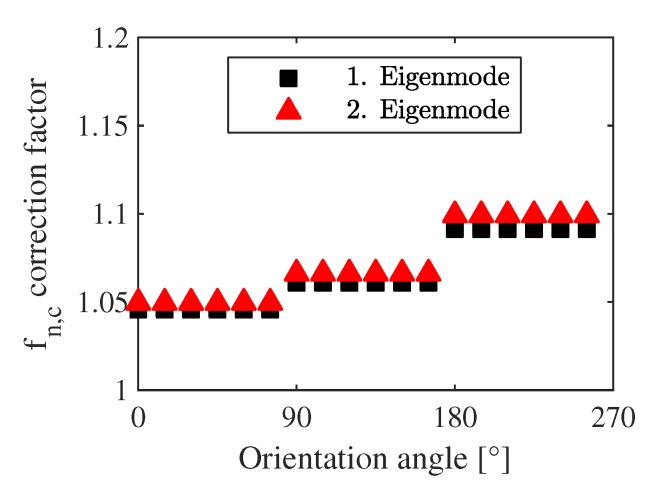
Undercut correction factor to apply on the cantilever eigenfrequencies for the first two eigenmodes. Different structure lengths (see Figure 1b)) are charged with different factors.

In theory, normalizing Equation (9) by the length and the eigenvalue should yield a constant value. However, this effect is not observed in the experimental data. In Figure 7, a decrease of $f_{n,c}$ is found when the cantilever length is reduced and thus a deviation from the expected constant is observed comparing to the EEBT. It was found, that this dependency can be described by the empirical fit function

(17) $$s_{n}=c_{1,n}\left(1-\frac{|c_{2,n}|}{l_{c}}\right),$$

with the presumed accordance to SEM images where the undercut is angle-independent after the isotropic etching in the fluorine plasma. In the limit $l_{c}\;\overset{}{\to }\;\infty$, the active undercut $u_{c}$ can be derived from:

(18) $$u_{c,n}=\left(1-c_{1,n}\right)l_{c},$$

which results in −4.56 μm and −4.96 μm for the first and second eigenmode, respectively. The estimated undercut is lower than the actual one (≈ 7.3 μm, determined by SEM), and it can be interpreted as the deflection of the cantilevers damped within the undercut region. The length of each cantilever is corrected by $u_{c}$ subsequently and further calculations are carried out with the adjusted eigenfrequencies for s- and h-structures (see Figure 8). As $u_{c}$ is independent of orientation, variations in the eigenfrequencies arising from the anisotropy of material properties are not changed.

**Figure 9:**
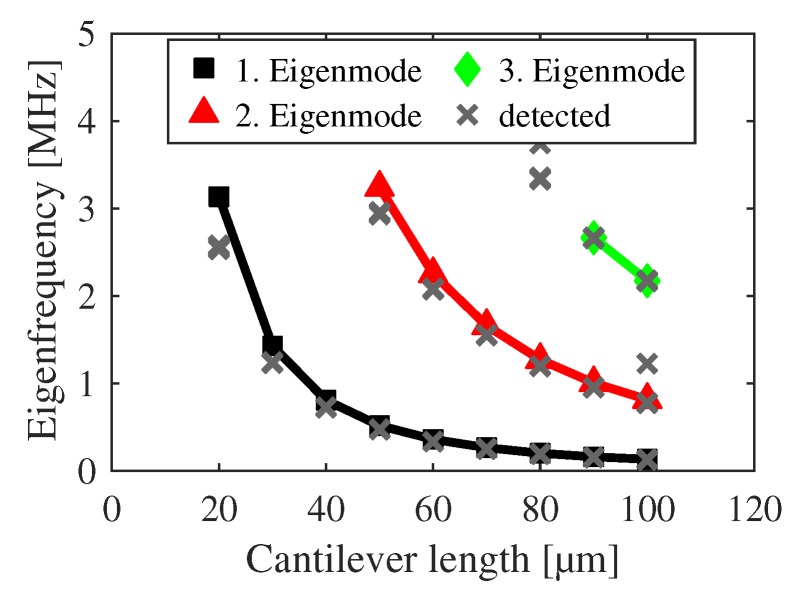
Classified and averaged eigenfrequencies of h-cantilevers in dependency on their length for the first three eigenmodes. Detected eigenfrequencies according to Section 3 are marked as crosses for comparison.

**Figure 10:**
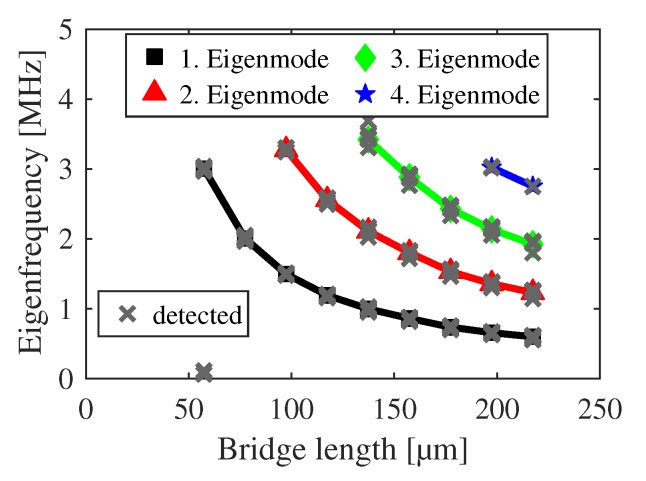
Classified and averaged eigenfrequencies of h-bridges in dependency on their length for the first four eigenmodes. Detected eigenfrequencies according to Section 3 are marked as crosses for comparison.

The classified eigenfrequencies of the h-structures are shown in Figure 9 and Figure 10 with a strictly monotonic decrease for increasing lengths. The plots serve as verification whether any averaging was successful or not. An eigenfrequency shift is observable in Figure 9 in relation to the detected modes according to Section 3, which arises from the undercut correction. Figure 10 shows additional eigenfrequencies at 60 μm which have been omitted during the averaging procedure. Additional diverging frequencies are out of scale. In total, 1057 of 1283 eigenfrequencies are used for further analysis.

As a first computational result, the strain $\epsilon_{b}$ can be derived directly from Equations (9) and (10) using the averaged eigenfrequencies [40] as

(19) $$\epsilon_{b}=\left({\left(\frac{f_{n,b}}{f_{n,c}}\right)}^{2}{\left(\frac{\kappa_{n,c}l_{b}}{\kappa_{n,b}l_{c}}\right)}^{4}-1\right)\frac{h^{2}}{\gamma_{n,b}l_{b}^{2}}.$$

Because the strain within a bridge is assumed to be a constant, the calculated is the averaged value of the respective structure. Actually, the strain exhibits a complex distribution along a bridge arising from temperature-dependent stresses during growth which are released while suspending the bridges. These stresses cannot be modeled within the EEBT, but the results of the gained strains can be used for comparison purposes with FEM simulations and their predictions. They are also a figure of merit of the final magnetoelectric sensor, e.g., for the determination of coupling factors between the magnetic field and the strain.

**Figure 11:**
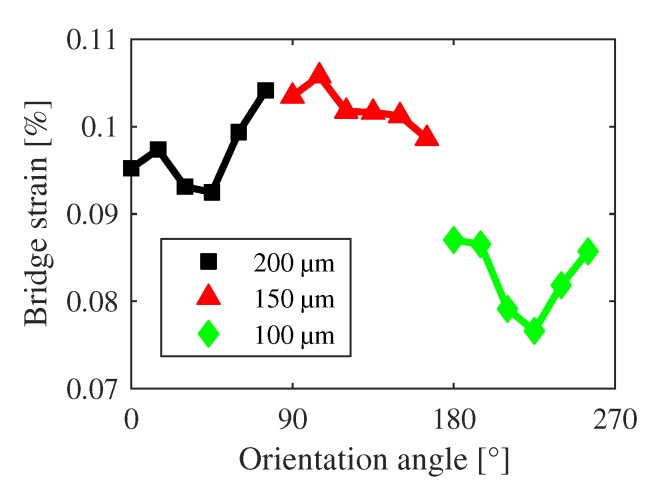
Average strain of the s-bridges in dependency on orientation angle and bridge length.

**Figure 12:**
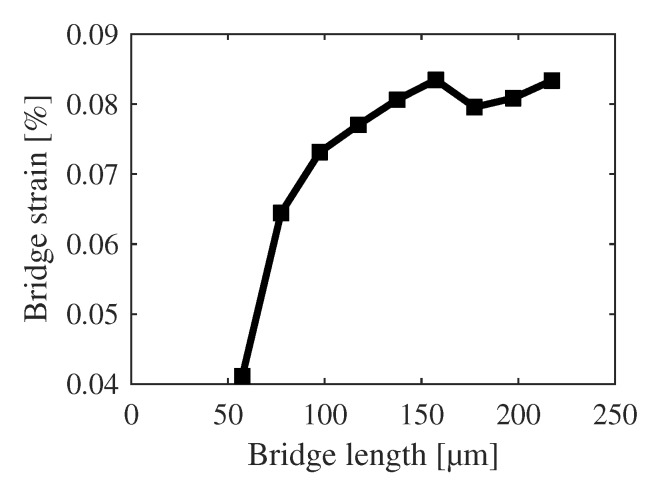
Average strain of the h-bridges in dependency on bridge length.

The strain itself is not only influenced by thermal stresses or the magnetic field, but also by the lattice mismatches and resulting dislocations at the ScAlN(0001)/Pt(111) and Pt(111)/Si(111) interfaces [44]. Figure 11 shows the calculated bridge strain as a function of the orientation angle where the structure lengths change with increasing angle in accordance to Figure 1a. Using Figure 12, it can be stated that the strain is length-dependent and smaller lengths exhibit smaller strains. The overlaying “oscillation” in Figure 11 is thus a pure effect of the structure orientation. The changing of the rotational and translational lattice mismatch between the polycrystalline ScAlN and Pt layers might be the possible explanation for this behavior. Nevertheless, the impact on the sensor performance is self-evident.

The determination of the strain as the first extracted parameter enables the calculation of the elastic modulus for the s- and h-structures, respectively. To keep the computational effort low, the eigenfrequencies are normalized by the constant parameters of Equation (10), which leads to

(20) $$\varsigma_{b}=\frac{f_{n,b}}{\sqrt{1+\gamma_{n}\frac{l_{b}^{2}}{h^{2}}\epsilon_{b}}}\frac{2\pi \sqrt{{\left(\rho A\right)}_{tot}}}{\kappa_{n,b}^{2}}=\frac{\sqrt{{\left(EI\right)}_{tot}}}{l_{b}^{2}}.$$

The trapezoidal shape of the ScAlN layer is considered in the cross section *A* and in the moment of inertia estimation. The parameters used for finding the Young’s modulus are shown in Table 2. The Young’s modulus of Pt is derived in a similar way. The angular variation of the Young’s modulus of Pt layer is about 1…2% due to its high polycrystallinity. In combination with the small thickness of about 80 nm, the influence on the ScAlN Young’s modulus is negligible and the fit is carried out using a constant value of 126.8 GPa.

The modal analysis is completed with the calculation of both parameters, strain and Young’s modulus for the s- and h-structures. The calculation time of a single analysis is on average about 0.5 s, which is a decisive advantage over manual analysis, which usually takes a few days for a comparable set of data.

## 7. Results

**Figure 13:**
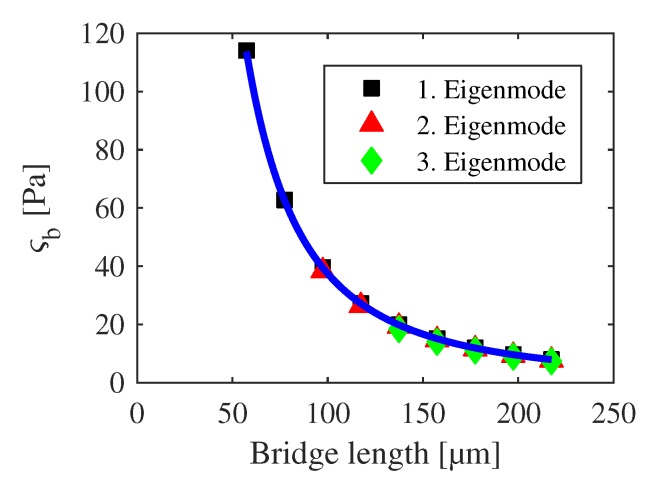
Eigenfrequencies of the h-bridges normalized by the constants of Equation (10) in dependency of the length. The slope of the fit function is proportional to the square root of the Young’s modulus.

**Figure 14:**
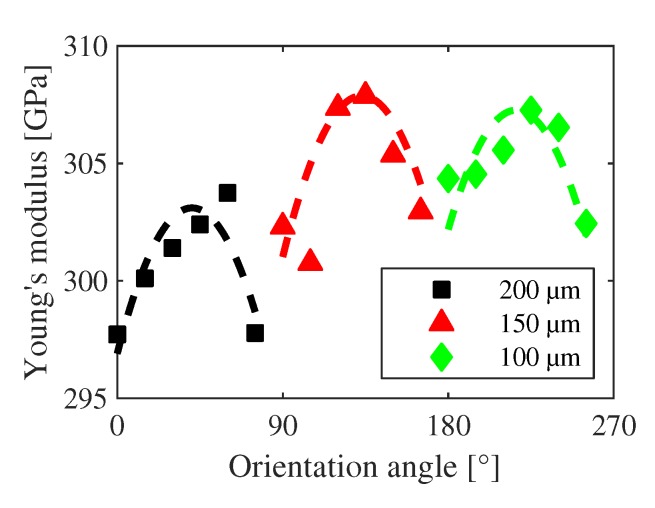
Calculated Young’s moduli for different orientation angles and lengths of the s-structures. Though the ScAlN is polycrystalline, an anisotropy in the modulus is verifiable for the different lengths. Decreasing Young’s modulus for increasing lengths indicates a size effect.

The Young’s modulus for the h-structures is determined to 315.2 GPa according to the fit in Figure 13. The accuracy of the fit is quite high, as fifteen eigenfrequencies for three (normalized) eigenmodes are used to derive the single value. For comparison, the s-structures have only three different lengths and thus fewer eigenfrequencies can be used in fitting, which leads in general to a higher uncertainty and scattering of the determined moduli (see Figure 14). Two effects can be observed by comparing the moduli for the different lengths of the structures. The first one is the angle-dependent anisotropy. The sputtered material with typical grain sizes less than 100 nm exhibits twisted pillars [45] with strong c-axis orientation, which can also be seen in their own X-ray diffraction (XRD) and atomic force microscopy (AFM) measurements. Thus, an isotropic Young’s modulus is expected in contrast to the calculated moduli. However, the achieved result is in agreement with theoretical investigations of the anisotropy of wurtzite AlN [46]. Though there is no epitaxial relationship between ScAlN, Pt and silicon, the use of (111) oriented Si leads to a weak preferential orientation of the (111) oriented Pt and the subsequent hexagonal ScAlN layer. The second effect is related to the seemingly dependency of the Young’s modulus on the structure length. The Young’s modulus appears to decrease for longer beam lengths while keeping a similar dependency on the anisotropy. This effect cannot be related to single deviating s-structures as all redundant beams exhibit the same eigenfrequencies within a small statistical deviation. Thus, external reasons of the apparent softening of long beams caused by cracks, particle load, or other local influences on the respective structures can be excluded. All bridges have the same width which excludes a possible size effect here. Instead, the reason for the seemingly higher Young’s modulus of shorter beams is assumed to be a visible effect of the deviation between the idealized structure used for calculation and the real doubly clamped beam. Though the influence of the technologically-caused undercut was taken into account in terms of an effective beam length, additional deviations at the clamping points cannot be considered in their full influence. The strain for example is taken to be constant across the beam length. A deviation in the area of the anchors due to a not fully relaxed region cannot be considered in the model presented here. These influences are strongest around the anchored region and negligible across the beam in its full length. Thus, they will increase the stiffness (hence the subsequently calculated Young’s modulus) of shorter beams and lose influence for longer ones. In addition, a possible change in the moment of inertia due to a partial relaxation and the formation of a weak rain-pipe-like cross section is not considered. Such an increase of the moment of inertia would lead to a decrease of the calculated Young’s modulus at a given eigenfrequency, especially considering the fact that the longest bridges exhibit the highest strains. In order to estimate the error of neglecting the actual strain profile, initial FEM simulations of 250 μm long bridges similar to the measured ones were carried out—one with fixed constraints to model a bridge according to the EEBT and one with undercut to model the real strain distribution. The results indicate that the error in the strain determination arising from thermal stresses during the growth at 300 ${}^{{}^{\circ}}C$ is about 1.3% with decreasing tendency for decreasing strains.

Table 3 summarizes the extracted Young’s moduli in comparison to literature values of ScAlN layers with a scandium amount between 5 and 15 at.-%. All literature values were measured by nanoindentation where anisotropy effects play a role but are apparently not considered [47]. This leads to significant uncertainties in the given values for the bulk-like layers. Incorporating scandium into the AlN lattice decreases the Young’s modulus as found by [25] and is also reproduced in the table. The variation in the anisotropy from this study is found to be within the range of the error bar given in the measurements of the cited literature, i.e., the resolution of the proposed method is higher than by nanoindentation unveiling an anisotropy effect hidden in the measurement uncertainty of the literature. Given the approximately parabolic dependency of the ΔE effect in the low magnetic field regime [18], an increased resolution in the determination of the Young’s modulus is highly beneficial for the analysis of the sensor performance. As a consequence, the proposed method avoids, for example, a false attribution of anisotropy related Young’s modulus shifts to changes in the magnetic field while characterizing a sensor. Additionally, the increased resolution enables investigating anisotropy effects in detail, especially when magnetostrictive layers are considered, where the material composition, texture, and the orientation of the magnetic domains play an important role.

## 8. Conclusions

In this work, a new method for the fully automated and fast parameter extraction for resonantly driven MEMS on the basis of a co-sputtered Pt/ScAlN layer system was proposed, which is applicable to MEMS based devices in general and particularly to magnetoelectric sensors. In particular, the latter ones demand high resolution of the measured parameters as several effects affect the frequency behavior of such a device, namely the ΔE effect, induced stresses or geometrical changes. The described method allows the determination of the Young’s modulus and the average strain of such structures while benefitting from an increased resolution compared to commonly used nanoindentation techniques. This was reached through the analysis of the eigenfrequency behavior depending on the length and orientation of singly and doubly clamped beams in two steps. Firstly, a new fit function was introduced as it could be shown that the often used Lorentzian function is not suitable for fitting eigenfrequency spectra. While the error in determining the eigenfrequency itself is negligible, the error of the extracted amplitude and FWHM can be up to 13% and 26% otherwise, respectively. However, higher deviations could also be observed which apply accordingly to derived parameters, e.g., the quality factor. To minimize fitting errors, a full fit of the eigenfrequency spectrum was carried out subsequently and the extracted eigenfrequencies of the respective structures were used to determine the Young’s modulus and the strain. This was realized in the second step using an extended Euler–Bernoulli-theory in an automated way. Simplifications within the Euler–Bernoulli-theory in terms of a constant strain-dependent eigenvalue $\gamma_{n}$ and a neglected complex strain profile introduce an error of about 1% each. An innovative and robust algorithm for automated classification of bending modes of cantilevers and bridges was developed. The extracted Young’s moduli were in the range between 298 - 315 GPa and thus comparable to values given in the literature, but more interestingly exhibit an angle-dependent anisotropy effect. This anisotropy was not shown before and varies within the uncertainty range of nanoindentation measurements. Accordingly, such measurements are lacking accuracy in determining specific characteristics, like the ΔE effect when omitting the anisotropy or other influences like geometry changes. Compared to the manual analysis of the measurement data containing 322 structures, the proposed automation is able to reduce the needed time significantly from approximately ten working days down to two hours.

## Figures and Tables

**Table 1:** Initial and optimized values for the two stage curve fit of the first eigenfrequency of the spectrum shown in Figure 3 and Figure 5.

	Initial Values	Initial Values for Joined Fit	Optimized Values
$a_{n}$	$1.3848\times 10^{-7}$ Hz $m^{-1}$	$1.1761\times 10^{-7}$ Hz $m^{-1}$	$1.0828\times 10^{-7}$ Hz $m^{-1}$
$p_{n}$	2.2198 k Hz	1.9044 k Hz	1.7316 k Hz
$f_{n}$	0.3556 M Hz	0.3558 M Hz	0.3558 M Hz
$\theta_{n}$	1.7104 p m	0.7549 p m	0.8392 p m

**Table 2:** Fit parameters used for the determination of the Young’s modulus. The density of scandium aluminium nitride (ScAlN) is derived from hexagonal lattice constants given in the reference.

Parameter	Platinum Layer	ScAlN Layer
Density	21,450 kg $m^{-3}$	3318 kg $m^{-3}$ [25]
Young’s modulus	126.8 G Pa	
Etching angle	0${}^{{}^{\circ}}$	25${}^{{}^{\circ}}$

**Table 3:** Comparison of the determined Young’s modulus of ScAlN with literature values of similar scandium concentrations including growth process and layer thickness. * Values are interpolated.

Material	Growth	Thickness	Young’s Modulus	Reference
Sc${}_{0.05}$Al${}_{0.95}$N	co-sputtered	(0.6–1) μm	(250–275) GPa	[48]
Sc${}_{0.08}$Al${}_{0.92}$N	co-sputtered	1 μm	(298–315) GPa	this work
Sc${}_{0.1}$Al${}_{0.9}$N	co-sputtered	0.45 μ m	(300–320) GPa	[25]
Sc${}_{0.1}$Al${}_{0.9}$N	co-sputtered	10 μm	290 * GPa	[49]
Sc${}_{0.15}$Al${}_{0.85}$N	co-sputtered	1 μm	200 GPa	[50]

## Supplementary Materials

The following are available online at https://www.mdpi.com/1424-8220/20/4/1001/s1.
